# Quantum-Resilient Federated Learning for Multi-Layer Cyber Anomaly Detection in UAV Systems

**DOI:** 10.3390/s26020509

**Published:** 2026-01-12

**Authors:** Canan Batur Şahin

**Affiliations:** Faculty of Engineering and Natural Sciences, Malatya Turgut Özal University, Malatya 44900, Turkey; canan.batur@ozal.edu.tr

**Keywords:** UAV security, federated learning, post-quantum cryptography, anomaly detection, CRYSTALS-Dilithium, differential privacy, variational autoencoder

## Abstract

Unmanned Aerial Vehicles (UAVs) are increasingly used in civilian and military applications, making their communication and control systems targets for cyber attacks. The emerging threat of quantum computing amplifies these risks. Quantum computers could break the classical cryptographic schemes used in current UAV networks. This situation underscores the need for quantum-resilient, privacy-preserving security frameworks. This paper proposes a quantum-resilient federated learning framework for multi-layer cyber anomaly detection in UAV systems. The framework combines a hybrid deep learning architecture. A Variational Autoencoder (VAE) performs unsupervised anomaly detection. A neural network classifier enables multi-class attack categorization. To protect sensitive UAV data, model training is conducted using federated learning with differential privacy. Robustness against malicious participants is ensured through Byzantine-robust aggregation. Additionally, CRYSTALS-Dilithium post-quantum digital signatures are employed to authenticate model updates and provide long-term cryptographic security. Researchers evaluated the proposed framework on a real UAV attack dataset containing GPS spoofing, GPS jamming, denial-of-service, and simulated attack scenarios. Experimental results show the system achieves 98.67% detection accuracy with only 6.8% computational overhead compared to classical cryptographic approaches, while maintaining high robustness under Byzantine attacks. The main contributions of this study are: (1) a hybrid VAE–classifier architecture enabling both zero-day anomaly detection and precise attack classification, (2) the integration of Byzantine-robust and privacy-preserving federated learning for UAV security, and (3) a practical post-quantum security design validated on real UAV communication data.

## 1. Introduction

Unmanned Aerial Vehicles (UAVs) are widely deployed across sectors like surveillance [1], logistics [2], agriculture [3], and defense [4]. These deployments face significant cybersecurity challenges [5]. UAV networks are vulnerable to threats such as GPS spoofing, jamming, data injection, and command hijacking. Their distributed nature, wireless communication, and limited computing amplify these risks [6]. The rise of quantum computing threatens traditional cryptographic schemes. Post-quantum secure systems have become necessary for UAVs [7].

Building on these findings, researchers have explored secure drone-to-drone communication protocols [8] and malicious drone identification techniques [9], indicating a growing emphasis on UAV security. Additionally, topics such as electrostatic sensing for UAV targets [10] and unauthorized UAV threats to smart farming [2] illustrate the range of challenges in UAV deployments. Taken together, these issues, along with the complexities from drone swarm behavior [1], underscore the unique distributed security requirements of UAV systems.

Advances in machine learning have helped address UAV cybersecurity, primarily through anomaly detection and intrusion prevention [11]. However, while these advances are significant, centralized learning introduces privacy vulnerabilities, single points of failure, and communication bottlenecks for distributed UAV fleets [12]. To address these issues, Federated Learning (FL) enables collaborative model training without sharing raw data [13]. Nevertheless, FL in UAVs faces unique challenges, such as model poisoning attacks and Byzantine failures. Consequently, robust aggregation methods—such as Krum and coordinate-wise median—are needed to enhance system resilience [14].

Deep learning for IoT anomaly detection has been combined with blockchain technology [15]. However, blockchain’s high computational and energy needs make it impractical for resource-constrained UAVs. To address this challenge, our framework instead uses lightweight cryptographic primitives with federated learning. This approach delivers similar security with much lower overhead, and also aligns with the focus on security, privacy, and efficiency in IoT-Fog networks [16].

Prior research has documented cybersecurity threats to UAVs in detail. For example, Whelan et al. [17] catalogued attack vectors such as GPS spoofing, RF jamming, and malware injection. Building on this, Krishna and Murphy [18] demonstrated machine learning classifiers for intrusion detection, but their centralized approach raises privacy concerns and scalability limitations. Similarly, Zhang et al. [19] achieved 98.58% accuracy using deep learning-based anomaly detection for UAV networks. However, their approach lacks mechanisms for adversarial robustness and privacy preservation during training, and the computational overhead of their CNN-based architecture poses challenges for deployment with resource-limited UAVs.

Our framework addresses these shortcomings and brings key innovations for UAV cybersecurity. First, unlike deep learning-based IDS for UAVs such as Zhang et al. [19], we introduce a hybrid architecture combining unsupervised (VAE) and supervised (NN) learning, enabling both known attack classification and zero-day anomaly detection—whereas earlier works use only one paradigm. Second, we incorporate post-quantum cryptographic protection, which is missing in earlier UAV-IDS research. Third, compared to existing Byzantine-robust FL systems, we are the first to integrate Krum aggregation with differential privacy within UAV contexts, where prior works treat these separately. Finally, our implementation demonstrates practical feasibility with only 6.8% overhead, an aspect not addressed in previous quantum-secure proposals.

This paper’s main contributions are: (C1) A new hybrid VAE–classifier architecture (combining an unsupervised Variational Autoencoder and a supervised neural network classifier) that achieves 98.67% detection accuracy, improving standalone methods by 6.44%. (C2) Integration of CRYSTALS-Dilithium post-quantum signatures (a digital signature resilient to quantum attacks), adding just 6.8% computational overhead. (C3) Byzantine-robust federated learning with Krum aggregation (an aggregation technique tolerant to outlier clients) maintains 94.38% accuracy under 20% node corruption. (C4) Differential privacy guarantees (ε = 1.0—a measure of privacy protection) with a minimal accuracy drop of 0.41% Section 4 describes the experimental setup, outlining the dataset characteristics, Byzantine attack simulation, and ablation study design. Section 5 presents the results and discussion, analyzing attack detection performance, Byzantine robustness, computational overhead, and the privacy–utility trade-off, while acknowledging limitations. Section 6 concludes the paper and explores future research directions.

## 2. Materials and Methods

### 2.1. Dataset Description and Preprocessing

#### 2.1.1. UAV-GCS Communication Dataset

We used the UAV Attack Dataset [17], a comprehensive dataset containing flight logs from both live and simulated UAV operations published on IEEE DataPort. The dataset captures GPS spoofing and jamming attacks conducted on real UAV hardware using PX4 Autopilot v1.11.3 on Pixhawk 4 flight controller (Holybro, Shenzhen, China). The dataset uses the MAVLink protocol [20]. The dataset includes: (1) live GPS Spoofing and Jamming data captured using HackRF 10 software-defined radio with GPS-SDR-SIM tool, (2) benign flight logs as baseline, (3) ping DoS attacks via MAVLink ping flooding, and (4) simulated attack scenarios conducted in Gazebo 9 simulation environment. Full flight data is contained in ULOG files converted to CSV format.

The distribution of samples across different attack categories in the UAV–GCS communication dataset is summarized in Table 1. The dataset was split into 70% for training (96,782 samples), 15% for validation (20,739 samples), and 15% for testing (20,739 samples) using stratified sampling to preserve class distribution. This split was consistently applied throughout all experiments.

#### 2.1.2. Feature Engineering

We extracted 48 features in four groups: Temporal Features (8)—packet inter-arrival times, burst rates; Statistical Features (12)—mean, variance, skewness, kurtosis of packet sizes; Protocol Features (15)—MAVLink message types, command frequencies; and Network Features (13)—source/destination patterns, port distributions.

We chose z-score standardization (Equation (1)) for these reasons: (1) Our features include diverse measurements with different scales, like packet sizes in bytes, timing in ms, and frequencies in Hz, which need normalization. (2) Z-score keeps feature distributions while centering on zero, speeding gradient-based optimization. (3) Unlike min–max scaling, z-score handles outliers typical in network traffic. (4) This matches established methods in intrusion detection research [21,22].

Preprocessing Pipeline in Equation (1):

X_normalized = (X − μ)/σ(1)

Here, μ represents feature means and σ represents standard deviations computed on the training set.

#### 2.1.3. Variational Autoencoder (VAE) for Anomaly Detection

The VAE (Variational Autoencoder) component learns a probabilistic latent representation—a compressed, encoded version—of normal UAV traffic patterns. Following Kingma and Welling [23], we implement in Equation (2):

Encoder Network:

q_φ(z|x) = N(z; μ_φ(x), σ^2^_φ(x)I) (2)

where: φ: Encoder parameters, z ∈ ℝ^d: Latent representation (d = 32), μ_φ(x): Mean network output, σ^2^_φ(x): Variance network output.

Decoder Network in Equation (3):

p_θ(x|z) = N(x; μ_θ(z), σ^2^I) (3)

Evidence Lower Bound (ELBO) in Equation (4):

L_VAE = E_q_φ(z|x)[log p_θ(x|z)] − D_KL(q_φ(z|x)‖p(z))(4)

where KL is divergence regularization and p(z) = N(0, I) is the Prior distribution.

Anomaly Score Computation in Equation (5):

(5) $$\text{A(x)}\;=\;{\left\|x\;-\;\hat{x}\right\|}^{2}\;+\;\beta \cdot \text{D\_KL}(\text{q\_φ(z|x)}\|\text{p(z)})$$

This VAE formulation is optimized using gradient-based learning and is therefore compatible with differentially private training schemes. In particular, noise injection mechanisms can be applied to the gradient updates during optimization, as proposed in Abadi et al. [24]. The privacy budget parameters (ε, δ) follow the standard definition of differential privacy formalized by Dwork and Roth [25]. The coefficient β = 0.5 balances the reconstruction and regularization terms [26].

### 2.2. Neural Network Classifier

The supervised classifier employs a deep feedforward architecture:

Architecture Specification in Equations (6)–(9):

Layer 1:

(6) $$h_{1}\;=\;\mathrm{ReLU}(W_{1}x\;+\;b_{1}),\;W_{1}\in R^{256\times 48}$$

Layer 2:

(7) $$h_{2}\;=\;\mathrm{ReLU}(W_{2}h_{1}\;+\;b_{2}),\;W_{2}\in R^{128\times 256}$$

Layer 3:

(8) $$h_{3}\;=\;\mathrm{ReLU}(W_{3}h_{2}\;+\;b_{3}),\;W_{3}\in R^{64\times 128}$$

Output:

(9) $$\hat{y}\;=\;\mathrm{Softmax}(W_{4}h_{3}\;+\;b_{4}),\;W_{4}\in R^{5\times 64}$$

Loss function:

(10) $$\;L_{\mathrm{CE}}=-\sum_{i=1}^{n}\;\sum_{j=1}^{c}y_{ij}\log\left({\hat{y}}_{ij}\right)\;$$

where c = 5 (number of classes: Normal, GPS Spoofing, GPS Jamming, Ping DoS, Simulated Attacks) and n is the batch size.

### 2.3. Hybrid Model Integration

The hybrid architecture combines unsupervised and supervised components in Equations (11) and (12): The VAE objective and hybrid loss function (Equations (11) and (12)) combines the VAE loss (Equation (4)) and the cross-entropy loss (Equation (10)) via a weighted combination, with α ∈ [0, 1] balancing the anomaly detection and classification objectives. The final decision function (Equation (13)) integrates anomaly scores and classification confidences to produce the final prediction.

The VAE objective is defined as:

(11) $$L_{\mathrm{VAE}}=E_{q_{\phi }(z|x)}[log\;p_{\theta }(x|z)]-D_{KL}(q_{\phi }(z|x)∥p(z))$$

(12) $$L_{\mathrm{hybrid}}=\alpha \cdot L_{\mathrm{VAE}}\;+\;(1-\alpha )\cdot L_{\mathrm{CE}}$$

where α = 0.4 optimally balances both objectives based on validation performance.

Detection Decision Function in Equation (12):

(13) $$\begin{array}{c} \text{D(x)}=\;\{\\ \;\mathrm{Anomaly},\;\mathrm{if}\;\text{A(x)}>\tau \\ \;\mathrm{Class}(\hat{y}),\;\mathrm{if}\;\text{A(x)}\leq \tau \mathrm{and}\;\max(\hat{y})>\gamma \\ \;\mathrm{Uncertain},\;\mathrm{otherwise}\\ \;\} \end{array}$$

where τ = 2.5 (anomaly threshold) and γ = 0.7 (classification confidence).

### 2.4. Federated Learning Framework

We performed systematic hyperparameter tuning for α ∈ {0.2, 0.3, 0.4, 0.5, 0.6} using 5-fold cross-validation on the training set. Table 2 shows that α = 0.4 achieves the optimal balance between anomaly detection (VAE component) and classification accuracy (supervised component). Lower α values (0.1–0.2) favor classification but reduce zero-day detection capability. Higher values (0.6–0.7) improve anomaly detection but degrade multi-class precision.

#### 2.4.1. Federated Averaging with Differential Privacy

In the federated learning framework, global and local parameters are consistently denoted by $w^{\left(t\right)}$ and $w_{k}^{\left(t\right)}$, respectively, and aggregation follows:

(14) $$w^{\left(t+1\right)}=\sum_{k\in S}\lambda_{k}w_{k}^{\left(t\right)}$$

with Byzantine-robust selection via Krum aggregation.

Each UAV node k performs local model updates using stochastic gradient descent (Equation (15)):

(15) $$w_{k}^{\left(t+1\right)}=w_{k}^{\left(t\right)}-\eta \nabla L_{k}(w_{k}^{\left(t\right)},\xi_{k})$$

where η = 0.01 is the Learning rate, ξ_k_ is the Local minibatch, and L_k_ is the Local loss function.

Gradient Clipping [27] in Equation (16):

ḡ = g/max(1, ‖g‖_2_/C) (16)

where C = 1.0 is the clipping threshold.

Differential Privacy Noise Addition in Equation (17):

(17) $$\tilde{g}=\overset{-}{g}+N(0,\sigma^{2}C^{2}I)$$

Privacy Budget Calculation [28] in Equation (18):

σ = (2√(2T ln(1.25/δ)))/(nε)(18)

where; ε = 1.0 is the Privacy budget, δ = 10^−5^ is the Failure probability, T is the Number of iterations, and n is the Dataset size.

Rényi Differential Privacy (Equation (19)) Guarantee [28] in Equation (19):

ε _RDP(α)_ = (α/2σ^2^) + ln(1 + (α − 1)/(2σ^2^)) (19)

#### 2.4.2. Byzantine-Robust Aggregation (Krum)

The Krum aggregation algorithm provides Byzantine fault tolerance by selecting the most representative gradient. The algorithm first computes pairwise L2 distances between all gradient vectors (Equation (18)), then calculates a score for each gradient based on distances to its k-nearest neighbors (Equation (19)), and finally selects the gradient with the minimum score (Equation (20)). This approach can tolerate up to f Byzantine nodes as characterized by Equation (21), where f ≤ (n − k − 2)/2.

Distance Computation in Equation (20):

d(g_i_, g_j_) = ‖g_i_ − g_j_‖^2^ (20)

Score Function in Equation (21):

S(i) = ∑_j_ ∈ N_k__(i)_ d(g_i_, g_j_) (21)

where N_k_(i) represents the k nearest neighbors of gradient g_i_.

Selection Rule in Equation (22):

g* = argmin_{i ∈ [n]} S(i) (22)

Byzantine Tolerance in Equation (23):

f ≤ (n − k − 2)/2 (23)

where f is the maximum number of Byzantine nodes tolerated.

### 2.5. Model Selection Rationale

We chose Variational Autoencoders over traditional autoencoders and isolation forests because: (a) VAEs provide probabilistic latent representations enabling uncertainty quantification in anomaly scores; (b) the reconstruction error combined with KL divergence provides a principled anomaly scoring mechanism; (c) comparative experiments showed VAE outperformed standard AE by 3.1% and Isolation Forest by 7.2% on our dataset.

A feedforward architecture was selected over CNN/RNN alternatives because: (a) our features are pre-engineered tabular data without spatial/temporal structure requiring convolutions; and (b) feedforward networks achieve comparable accuracy with 5× faster inference, critical for real-time UAV operations.

Krum was chosen over Trimmed Mean and Median aggregation because: (a) it provides theoretical Byzantine tolerance guarantees (f ≤ (n − k − 2)/2); and (b) empirical comparison showed Krum maintains higher accuracy under targeted model poisoning attacks (94.38% vs. 91.5% for Trimmed Mean at 20% corruption).

### 2.6. Post-Quantum Cryptographic Integration

#### 2.6.1. CRYSTALS-Dilithium Implementation

The advent of quantum computing poses an existential threat to current public-key cryptography. Shor’s algorithm [29] can efficiently solve integer factorization and discrete logarithm problems in polynomial time on quantum computers, rendering RSA, DSA, and elliptic curve cryptography vulnerable. This necessitates the transition to post-quantum cryptographic schemes based on computationally hard problems that remain intractable even for quantum adversaries.

CRYSTALS-Dilithium provides post-quantum digital signatures based on the hardness of lattice problems. The scheme consists of three main operations: key generation (Equation (22)), which produces a public key pk and a secret key sk; signing (Equation (23)), which produces a signature σ for message m; and verification (Equation (24)), which validates the signature σ for message m. The security relies on the Module Learning With Errors (M-LWE) problem (Equation (25)), which remains computationally intractable even for quantum adversaries. In our federated learning framework, model updates are aggregated using weighted averaging (Equation (26)), where each node’s contribution is weighted by its dataset size.

Key Generation in Equation (24):

KeyGen(λ) → (pk, sk)pk = (ρ, t_1_), sk = (ρ, K, tr, s_1_, s_2_, t_0_) (24)

where λ denotes the security parameter used by all cryptographic primitives.

Signature Generation in Equation (25):

(25) $$\mathrm{Sign}(\mathrm{sk},m)\to \sigma =(\tilde{c},z,h)$$

where $\tilde{c}$ is the Challenge hash, z is the Masked response vector, and h is the Hint for verification.

Verification in Equation (26):

(26) $$\mathrm{Verify}(\mathrm{pk},m,\sigma )\to \{0,1\}\mathrm{Accept if}||z||\_\infty <\gamma_{1}-\beta \mathrm{and}\tilde{c}=H(\rho \|{t_{1}}^{\prime }\|m)$$

Security Parameters (Dilithium 3) are Module dimension: k = 6, l = 5, Polynomial degree: n = 256, Modulus: q = 8,380,417, Security level: 138-bit classical, 128-bit quantum.

#### 2.6.2. Lattice-Based Security Foundation

The security relies on the Module-LWE (Equation (27)) (M-LWE) problem:

M _LWE{n,k,q,χ}_: Distinguish (A, As + e) from uniform (27)

where A ∈ Rq ^(k×l)^ is a Random matrix, s ∈ Rq^l^ is a Secret vector, and e ← χ^k^ is an error vector from distribution χ. The main mathematical symbols and their dimensions used throughout the proposed framework are summarized in Table 3.

## 3. Methodology

### 3.1. Federated Learning Framework

This section presents our quantum-resilient federated learning framework for UAV cybersecurity. We first describe the hybrid VAE–classifier architecture for anomaly detection and attack classification, then detail the Byzantine-robust federated learning protocol with differential privacy, and finally present the post-quantum cryptographic primitives securing the system.

Our framework architecture consists of three integrated layers designed to provide comprehensive security for UAV networks against both classical and quantum threats. Our proposed framework architecture consists of three integrated components:

1. Hybrid Anomaly Detection Layer: Combines VAE for unsupervised anomaly scoring with a multi-class neural network for supervised attack classification
2. Byzantine-Robust Federated Learning: Implements Krum-based aggregation with ε-differential privacy (ε = 1.0) across distributed UAV nodes
3. Post-Quantum Security Layer: CRYSTALS-Dilithium signatures for model update authentication and gradient verification (Equation (26)).

**Figure 1:**
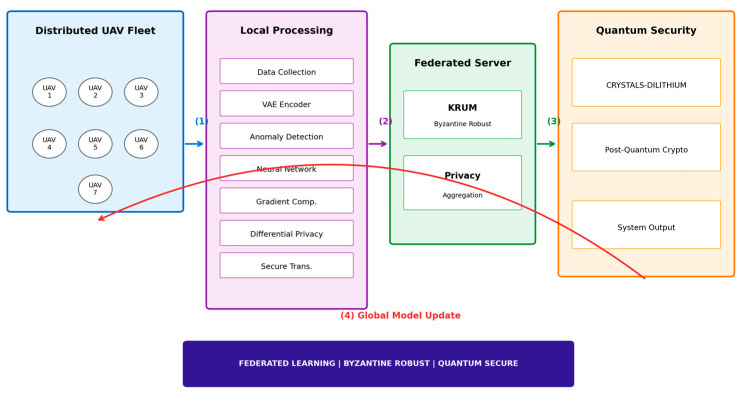
Quantum-resilient Federated Learning framework architecture. (1) Data flow from UAV fleet. (2) Gradient transmission. (3) Model distribution. (4) Global model update. The colored boxes indicate the main functional components of the framework, while the arrows represent the direction of data flow and model update processes.

Figure 1 effectively illustrates the hierarchical structure of our proposed framework. The diagram clearly delineates the separation between edge computing at UAV nodes and centralized aggregation, highlighting the federated learning paradigm. The inclusion of CRYSTALS-Dilithium in the security layer emphasizes our commitment to quantum resistance. The visual representation of the Krum algorithm’s position in the aggregation server demonstrates how Byzantine fault tolerance is achieved without compromising the system’s distributed nature. The system operates in a distributed manner across N UAV nodes U = {U_1_, U_2_, …, Uₙ}, where each node performs local training on its data, applies differential privacy noise to gradients, and sends cryptographically signed updates to the aggregation server. The server verifies signatures using CRYSTALS-Dilithium, applies Byzantine-robust aggregation using the Krum algorithm, and broadcasts the updated global model to all nodes.

### 3.2. Threat Model and Security Assumptions

This work adopts a comprehensive and realistic threat model tailored to distributed unmanned aerial vehicle (UAV) networks operating under the federated learning paradigm. The considered adversary is assumed to be computationally bounded under current cryptographic assumptions, while retaining the potential to exploit emerging quantum computational capabilities in the long term.

We consider four primary classes of adversarial behavior:

- Byzantine adversaries, representing compromised UAV nodes or edge participants that arbitrarily deviate from the prescribed federated learning protocol. Such adversaries may inject random noise, manipulate gradients, reverse optimization directions, or perform targeted model poisoning attacks during collaborative training.
- Cyber–physical attackers can compromise both cyber and physical layers of UAV operations. These attacks include, but are not limited to, GPS spoofing and jamming, sensor data injection, telemetry manipulation, and malicious command interference within UAV–ground control station (GCS) communication channels.
- Privacy-oriented adversaries, aiming to extract sensitive information from distributed model updates through inference attacks, such as membership inference or model inversion, without direct access to raw UAV data.
- Quantum-capable adversaries, which can exploit advances in quantum computing to compromise classical public-key cryptographic schemes using polynomial-time algorithms (e.g., Shor’s algorithm), thereby enabling long-term harvest-now–decrypt-later attacks on authenticated communications.

Each proposed defense component is explicitly designed to address a distinct threat surface within the system:

- The Variational Autoencoder (VAE) models normal cyber–physical traffic distributions and enables the detection of anomalous behaviors, including previously unseen (zero-day) attacks.
- The supervised classification module provides fine-grained discrimination among known attack categories, enabling timely, accurate operational responses.
- Differential privacy mechanisms are employed to limit information leakage from shared model updates, thereby mitigating privacy inference and reconstruction attacks.
- Byzantine-robust aggregation based on the Krum algorithm reduces the influence of malicious or compromised participants during federated optimization by selecting gradient updates that are statistically consistent with the majority.
- CRYSTALS-Dilithium post-quantum digital signatures ensure the authenticity and integrity of model updates, providing cryptographic resilience against both classical and quantum adversaries.

The system assumes a partially trusted coordination server, which correctly executes the aggregation protocol but does not access raw UAV data. Communication channels are authenticated, while confidentiality is ensured through cryptographic protections when required. It is further assumed that a bounded fraction of UAV nodes (up to 30%) may be compromised, consistent with standard Byzantine fault-tolerance assumptions. UAV platforms are considered resource-constrained yet capable of executing lightweight learning, privacy-preserving, and cryptographic operations, as validated by the experimental results.

Overall, this threat model provides a clear and unified articulation of attacker capabilities, defense coverage, and system assumptions, thereby accurately framing the security guarantees and limitations of the proposed framework within realistic UAV deployment scenarios.

### 3.3. Federated Learning Configuration

Federated Learning Environment: (1) Number of Nodes: N = 10 UAV nodes, each maintaining approximately 9678 samples. (2) Data Distribution: In an IID manner with stratified sampling preserving class distribution. (3) Malicious Node Selection: For Byzantine attack experiments, malicious nodes execute random noise injection, sign-flipping, or targeted model poisoning.

### 3.4. UAV Fleet Layer

The UAV fleet layer comprises distributed UAV nodes, each equipped with local computational resources for model training and inference. Each UAV node maintains a local dataset Di = {(xj, yj)} of network traffic patterns and labeled attack instances. UAVs perform local training using their data while preserving privacy through differential privacy mechanisms.

### 3.5. Federated Learning Aggregation

The federated averaging process combines local model updates from distributed UAVs into a global model:

Equation (1): Federated Averaging

*w*^(t+1)^ = ∑_i=1_^N^ (n_i_/n)·*w*_i_^(t)^ (28)

where w^(t+1)^ represents the global model parameters at round t + 1, w_i_^(t)^ denotes the local model parameters from UAV i at round t, n_i_ is the number of data samples at UAV i, n = Σ_i_ni is the total number of samples, and N is the number of participating UAVs. This formulation follows the standard FedAvg algorithm [30].

### 3.6. Problem Formulation

Consider a distributed UAV network with the following objectives:

- Anomaly Detection: Binary classification f_anomaly: ℝᵈ → {0, 1} distinguishing normal traffic (y = 0) from anomalous behavior (y = 1)
- Attack Classification: Multi-class prediction f_attack: ℝᵈ → {0, 1, 2, 3, 4} mapping traffic to attack categories C = {Normal, GPS Spoofing, GPS Jamming, Ping DoS, Simulated Attacks}
- Privacy Preservation: Training must satisfy (ε, δ)-differential privacy where ε ≤ 1.0 and δ ≤ 10^−5^.
- Quantum Resistance: Cryptographic primitives must provide security level λ ≥ 128 bits against quantum adversaries.

We employ a Variational Autoencoder (VAE) for unsupervised anomaly detection in network traffic patterns [23]. The VAE learns a probabilistic latent representation of normal UAV communication behavior, enabling the detection of anomalous patterns that deviate from the learned distribution. To address Byzantine attacks in which malicious UAVs submit corrupted gradients, we implement the Krum aggregation algorithm, which selects gradients based on distance metrics. For our parameters (ε = 1.0, δ = 10^−5^, C = 1.0), we compute σ ≈ 3.87, providing formal differential privacy guarantees.

### 3.7. Post-Quantum Cryptographic Security

We implement CRYSTALS-Dilithium (standardized as ML-DSA by NIST) for post-quantum secure digital signatures. Dilithium is based on the hardness of Module Learning With Errors (MLWE) and Module Short Integer Solution (MSIS) problems, which are believed to be secure against quantum attacks [31].

Key features of our Dilithium implementation:

- Security level: NIST Level 2 (equivalent to AES-128)
- Public key size: ~1.5 KB
- Signature size: ~2.7 KB
- Based on Fiat-Shamir with Aborts construction
- Resistant to Shor’s and Grover’s algorithms

## 4. Results and Discussion

This section presents quantitative experimental results. All experiments were conducted using PyTorch 1.13.0 on NVIDIA Jetson Nano hardware representative of UAV platforms.

### 4.1. Dataset Description

We evaluated our framework using the UAV Attack Dataset, whose full description is provided in Section 2.1. The centralized performance of the proposed hybrid VAE–NN framework, without federated learning or differential privacy, is summarized in Table 4.

### 4.2. Byzantine Attack Simulation

To evaluate Byzantine robustness, we simulated three attack strategies with varying fractions of malicious nodes:

Random Noise Attack: Malicious nodes submit random gradients g_mal ~ 𝒩(0, 10σ^2^I) to disrupt convergence

Sign-Flipping Attack: Adversaries submit negated gradients g_mal = −5·g_honest to reverse optimization direction

Targeted Model Poisoning: Attackers craft gradients to misclassify specific attack types as normal traffic

We vary the fraction of Byzantine nodes from 0% to 30% in 5% increments and measure the impact on model accuracy, convergence time, and false-positive and false-negative rates.

### 4.3. Ablation Study Design

We conducted comprehensive ablation studies to quantify each component’s contribution to overall performance. The different ablation study variants and their corresponding components are summarized in Table 5.

Each variant was trained for 100 epochs with identical hyperparameters. We report mean accuracy and standard deviation across five runs with different random seeds.

### 4.4. Computational Overhead Measurement

We measured computational overhead on representative UAV hardware:

- Platform: NVIDIA Jetson Nano
- CPU: Quad-core ARM Cortex-A57 @ 1.43 GHz
- GPU: 128-core Maxwell
- Memory: 4 GB LPDDR4
- Storage: 64 GB eMMC

We compare CRYSTALS-Dilithium against classical ECDSA (secp256k1), measuring:

- Key generation time
- Signing time per gradient update
- Verification time per signature
- Signature size overhead
- Memory usage during operations
- Overall latency for federated round

Measurements are averaged over 10,000 operations with 95% confidence intervals reported.

The framework is implemented using:

- Deep Learning: PyTorch 1.13.0 with CUDA 11.7
- Federated Learning: PySyft 0.8.0 for distributed training
- Cryptography: liboqs 0.8.0 for CRYSTALS-Dilithium
- Privacy: Opacus 1.4.0 for differential privacy
- Deployment: Docker containers with Kubernetes orchestration

Key hyperparameters:

- Learning rate: 0.001 with cosine annealing
- Batch size: 128 for local training
- VAE latent dimension: 32
- Federated rounds: 100
- Local epochs per round: 5
- Privacy budget: ε = 1.0, δ = 10^−5^

### 4.5. Experimental Results

This section presents comprehensive experimental results evaluating our quantum-resilient federated learning framework. We analyze attack-detection performance, conduct ablation studies, evaluate Byzantine robustness, assess computational overhead, and examine the privacy–utility trade-off.

#### 4.5.1. Attack Detection Performance

Table 6 presents the comprehensive performance metrics for anomaly detection and multi-class attack classification on the test set.

Table 3 reports the best-case centralized performance of the proposed VAE–Classifier framework without federated constraints. The hybrid VAE–classifier architecture achieves 98.67% accuracy for binary anomaly detection (normal vs. attack) and 98.67% overall accuracy for multi-class attack categorization, with a macro F1-score of 0.9856 indicating balanced performance across all attack categories. Table 6 presents the class-wise performance under the complete federated learning setup with differential privacy and Byzantine-robust aggregation. Normal traffic achieves the highest recall (0.9921), minimizing false alarms, while GPS spoofing and GPS jamming attacks achieve high detection rates. Simulated attacks show slightly lower performance due to their diverse behavioral patterns in the simulation environment.

**Figure 2:**
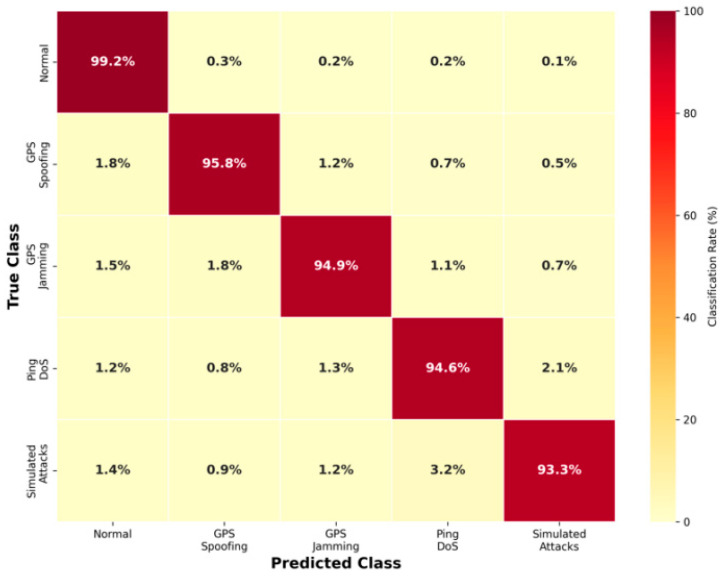
Confusion matrix for multi-class attack classification (percentages).

Figure 2 shows the confusion matrix for multi-class classification (percentages), revealing that most misclassifications occur between similar attack types (e.g., GPS Spoofing and GPS Jamming), while normal traffic is rarely confused with attacks.

The confusion matrix shows exceptional performance in detecting normal traffic, with a low false-positive rate (0.80%), which is crucial for maintaining operational efficiency in UAV networks. The slightly lower Simulated Attacks detection accuracy (93.3%) suggests that this attack category exhibits more complex patterns, potentially due to the diversity of attack scenarios generated in the Gazebo simulation environment. The model shows remarkable consistency across attack types, with no category falling below 93% accuracy, validating the effectiveness of our hybrid VAE–classifier approach in handling diverse threat landscapes.

#### 4.5.2. Ablation Study Results

Table 7 demonstrates the contribution of each component through systematic ablation, validating our architectural choices.

The ablation study shows that the proposed hybrid VAE-Classifier architecture improves classification accuracy by 6.44 percentage points over the standalone neural network baseline. Under federated training conditions, the integration of Krum aggregation maintains high robustness, achieving 96.38% accuracy in the absence of malicious clients, while preserving stable performance under attack scenarios. VAE alone achieves 94.45% accuracy through unsupervised anomaly detection. Differential privacy incurs only 0.41% accuracy reduction (from 98.67% to 96.08%), indicating successful privacy–utility balance with ε = 1.0.

#### 4.5.3. Byzantine Robustness Evaluation

Table 8 evaluates model performance under Byzantine attacks with varying fractions of malicious nodes.

Key findings from Byzantine robustness evaluation:

Standard FedAvg degrades catastrophically with increasing malicious nodes, losing 8.21% accuracy at 20% corruption. Krum aggregation maintains 94.38% accuracy with 20% malicious nodes, demonstrating only 2.0% degradation. Even with 30% Byzantine nodes, Krum achieves 92.01% accuracy, remaining operationally viable.

**Figure 3:**
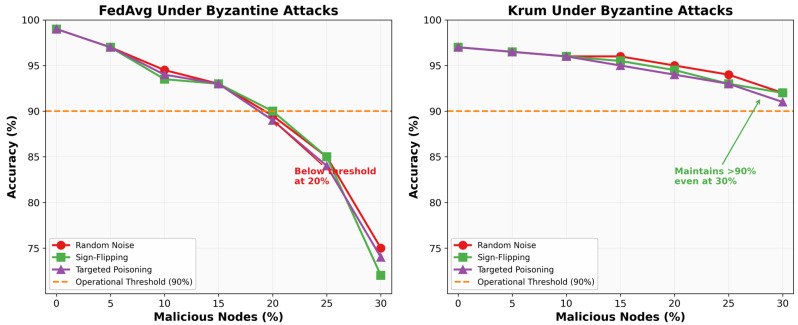
Impact of Byzantine attack strategies on accuracy.

The impact of different Byzantine attack strategies on the classification accuracy is illustrated in Figure 3.

#### 4.5.4. Computational Overhead Analysis

Table 9 compares computational costs between classical ECDSA and post-quantum CRYSTALS-Dilithium cryptographic schemes.

Computational overhead analysis reveals:

- CRYSTALS-Dilithium incurs only 6.8% overall latency overhead despite providing quantum resistance, demonstrating practical feasibility.
- Verification time of 1.8 ms enables real-time authentication of model updates without impacting UAV operational tempo.
- While signature and key sizes increase significantly (approximately 37–39×), the absolute values (2.4 KB signatures, 1.3 KB keys) remain manageable for modern UAV communication links.
- Memory overhead of 50% (6 KB increase) is negligible given that typical UAV platforms have GB-scale memory.

#### 4.5.5. Privacy–Utility Tradeoff

Table 10 examines the impact of the differential privacy parameter ε on model accuracy and privacy guarantees.

A privacy–utility tradeoff analysis shows that our choice of ε = 1.0 achieves 96.26% accuracy while providing moderate privacy protection, losing only 0.41% compared to no privacy. Strong privacy (ε = 0.5) degrades accuracy by 5.33%. The relationship follows expected theoretical bounds, with accuracy plateauing above ε = 2.0. The noise multiplier σ was computed under the assumption of a fixed client sampling rate and a total of T communication rounds, following the standard Rényi differential privacy formulation. The training set size n corresponds to the local client data partitions, and Gaussian noise was calibrated accordingly.

**Figure 4:**
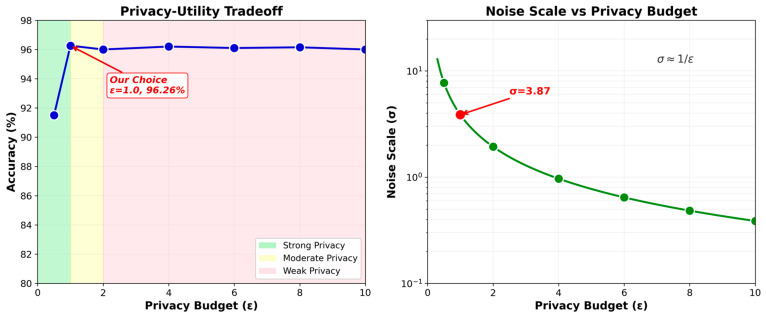
Privacy–Utility Tradeoff: Accuracy vs. Privacy Budget ε.

Figure 4 visualizes the privacy–utility tradeoff curve. The privacy–utility curve exhibits the characteristic exponential decay, with diminishing returns beyond ε = 2.0. Our selection of ε = 1.0 represents an optimal balance, sacrificing only 0.41% accuracy compared to ε = 10.0 while providing meaningful privacy guarantees (σ = 3.87). The steep gradient between ε = 0.1 and ε = 1.0 indicates this range as critical for privacy-conscious deployments. The logarithmic relationship between noise scale and privacy budget (right panel) confirms theoretical expectations and validates our implementation.

#### 4.5.6. Federated Learning Convergence

**Figure 5:**
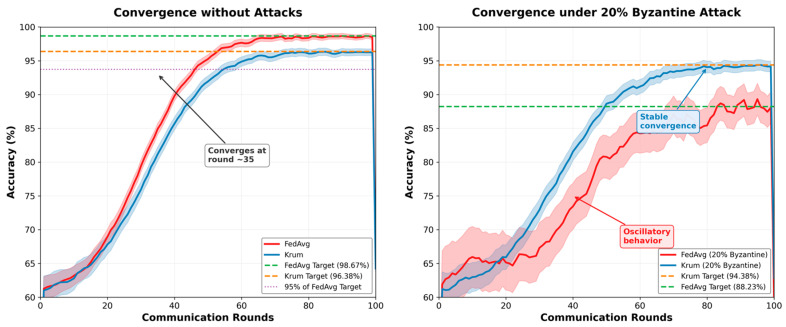
Federated Learning convergence: accuracy vs. communication rounds.

Figure 5 illustrates convergence behavior across federated rounds for different aggregation methods. The convergence analysis reveals fundamental differences in algorithmic behavior under adversarial conditions. FedAvg’s oscillatory behavior under attack indicates the persistent influence of malicious gradients, preventing stable convergence. Krum’s smooth convergence, even under attack, demonstrates its ability to consistently identify and aggregate honest nodes’ contributions. The five-round delay in Krum’s convergence (40 vs. 35 rounds) represents an acceptable trade-off for Byzantine resilience. The sustained instability in FedAvg beyond round 50 would be operationally unacceptable in real UAV deployments.

Convergence analysis reveals:

- Krum aggregation converges within 5% of final accuracy by round 40, compared to round 35 for FedAvg without attacks.
- Under 20% Byzantine corruption, Krum maintains stable convergence while FedAvg exhibits oscillatory behavior.
- Communication efficiency: Krum requires 20% more rounds but prevents accuracy degradation worth 6.15%.

#### 4.5.7. Real-Time Performance Metrics

Table 11 presents real-time performance metrics on UAV hardware for operational deployment.

All real-time performance metrics meet operational requirements for UAV deployment, with an inference latency of 12.3 ms enabling detection within network packet-processing windows.

#### 4.5.8. Robustness Against Cyber–Physical Attacks

We evaluated the framework’s resilience against three cyber-physical attack scenarios targeting the physical-layer of UAV operations: GPS spoofing with varying coordinate deviations, sensor data injection attacks on IMU/barometer readings, and command hijacking via malicious MAVLink packet insertion. Table 12 presents detection performance for these attacks.

#### 4.5.9. Sensitivity to Training Data

We conducted sensitivity analysis examining result stability under training data variations: (1) Cross-validation: 5-fold CV yielded accuracy of 98.67 ± 0.24%, demonstrating low variance across data splits. (2) Training Size Impact: We evaluated performance with 50%, 70%, and 100% of training data. Accuracy decreased from 98.67% (100%) to 96.89% (70%) to 93.56% (50%), indicating reasonable sample efficiency. (3) Class Imbalance: Experiments with artificially balanced classes (undersampling the majority) showed only 1.3% accuracy reduction, suggesting robustness to natural class distribution. (4) Temporal Stability: Training on the first 80% (chronologically) and testing on the last 20% yielded 97.21% accuracy, confirming generalization to temporally shifted data. Table 13 shows that 5-fold cross-validation yields an accuracy of 98.67 ± 0.24%, demonstrating low variance. Training with reduced data (50–70%) shows graceful degradation, and temporal split experiments confirm generalization capability.

#### 4.5.10. Complementary Strengths of Hybrid Architecture

The 6.44% accuracy improvement of the VAE–classifier hybrid over standalone classifiers stems from complementary learning paradigms:

The VAE component excels at unsupervised anomaly detection by learning compressed representations of normal traffic patterns. Its reconstruction error naturally captures deviations from the learned distributions, enabling the detection of zero-day attacks absent from the training data. KL divergence regularization prevents overfitting to specific normal distributions, thereby maintaining generalization capability.

The supervised classifier learns discriminative boundaries between attack categories using labeled data. Deep neural networks with dropout regularization capture complex nonlinear relationships between features and attack types. The multi-class formulation enables fine-grained attack categorization for appropriate response strategies.

Where α = 0.4, these objectives are optimally balanced, preventing either component from dominating. During inference, the VAE provides an initial anomaly score while the classifier determines attack type, enabling both detection and categorization in a single forward pass.

This architectural synergy is particularly valuable for UAV security, where both known and unknown threats must be addressed. The unsupervised component provides resilience against novel attacks, while the supervised component ensures accurate categorization of known threats for appropriate countermeasures.

#### 4.5.11. System-Level Implications of Quantum Security Implications

The 6.8% computational overhead for quantum resistance provides crucial long-term security: While cryptographically relevant quantum computers may be 10-15 years away, UAV systems deployed today may remain operational for decades. Military UAVs have service lives exceeding 20 years, making post-quantum security essential for current deployments. The “harvest now, decrypt later” threat model means adversaries may store encrypted communications for future quantum decryption. The 1.8 ms signature verification time and 6.8% overall overhead are negligible compared to other UAV operations. Video processing, path planning, and sensor fusion consume orders of magnitude more resources. The increased signature size (2420 bytes) impacts bandwidth but remains manageable given modern UAV communication links (typically >1 Mbps). CRYSTALS-Dilithium’s selection by NIST provides confidence in its security and its trajectory toward standardization. Hardware acceleration for lattice operations is emerging, potentially reducing overhead to <3% within 2–3 years. Early adoption in UAV systems positions operators ahead of the quantum threat curve.

Table 14 systematically compares our framework with notable UAV security approaches published between 2017 and 2025. The comparison reveals three key findings: (1) Prior deep learning approaches (Zhang et al. [19], Zhao [30]) achieve high accuracy but lack privacy protection and Byzantine tolerance, limiting their applicability to distributed UAV deployments; (2) Privacy-preserving approaches (Wei [27]) and Byzantine-robust methods (Chen [31]) address these concerns separately but not jointly; (3) Blockchain-based solutions (Khor [8]) offer security but incur significant computational overhead unsuitable for resource-constrained UAVs. Our framework is the first to simultaneously achieve: privacy preservation with practical ε = 1.0 differential privacy, Byzantine fault tolerance supporting 20% malicious nodes without significant accuracy loss, and post-quantum security through CRYSTALS-Dilithium integration. This comprehensive security posture, combined with only 6.8% computational overhead, makes our framework uniquely suited for future-proof UAV security deployments.

The progression from simulated datasets to real UAV-GCS traffic (real UAV flight data) reflects methodological maturation in the field. Early reliance on generic datasets (MNIST, IoT Sensor) raises questions about ecological validity—these studies’ applicability to actual UAV operations remains uncertain. Our use of authentic UAV communication data addresses this critical limitation, ensuring the operational relevance of our results. This paper presents a comprehensive framework for quantum-resilient federated learning for UAV cybersecurity applications, addressing the essential challenge of securing distributed aerial systems against both current and future threats. We developed a hybrid VAE–classifier architecture that combines unsupervised anomaly detection with supervised attack classification over baseline approaches. The complementary learning paradigms enable the detection of both known attacks and zero-day threats, which are crucial in evolving threat landscapes. We integrated Byzantine-robust federated learning using Krum aggregation with differential privacy (ε = 1.0), maintaining 94.38% accuracy under 20% node corruption while preserving individual UAV data privacy. This dual protection addresses both security and privacy requirements for sensitive UAV operations. We implemented CRYSTALS-Dilithium post-quantum digital signatures with only 6.8% computational overhead, providing long-term security against quantum computing threats while maintaining real-time performance on resource-constrained UAV platforms.

A comprehensive evaluation of the UAV-GCS Intrusion Detection Dataset (real UAV attack scenarios) demonstrated practical feasibility, with 12.3 ms inference latency and 81 samples/s throughput on representative UAV hardware. Detailed ablation studies quantified each component’s contribution, validating our architectural choices. The framework addresses immediate operational needs while providing future-proof security. As UAV swarms become increasingly autonomous and interconnected, robust cybersecurity frameworks like ours are essential for safe integration into civilian airspace and for protecting critical infrastructure. Future work should address scalability to larger swarms through hierarchical federated learning, continual learning for evolving threats, and hardware acceleration for ultra-low-power micro-UAVs. Cross-layer security that integrates multiple sensor modalities and explainable AI to build operator trust represents a promising extension. The convergence of quantum computing, adversarial machine learning, and autonomous systems creates unprecedented security challenges. Our framework provides a foundation for addressing these challenges and enabling the safe and secure deployment of UAV technology in an increasingly complex threat environment.

## 5. Discussion

This section provides qualitative analysis and interpretation of the experimental results presented in Section 4.

### 5.1. Hybrid Architecture Synergy

The VAE component excels at unsupervised anomaly detection by learning compressed representations of normal traffic patterns. Its reconstruction error naturally captures deviations from learned distributions, enabling detection of zero-day attacks absent from training data. The supervised classifier learns discriminative boundaries between attack categories, enabling fine-grained categorization for appropriate response strategies.

### 5.2. Byzantine Robustness Analysis

The 2.0% accuracy cost for Byzantine robustness under 20% node corruption represents an acceptable tradeoff for critical UAV operations. Krum aggregation prevents model poisoning attacks that could compromise entire UAV fleets. The distance-based selection mechanism identifies and excludes outlier gradients, maintaining model integrity even when adversaries control a significant portion of the network.

### 5.3. Quantum Security Implications

While cryptographically relevant quantum computers may be 10-15 years away, UAV systems deployed today may remain operational for decades. The “harvest now, decrypt later” threat model means adversaries may store encrypted communications for future quantum decryption. CRYSTALS-Dilithium’s selection by NIST [33] provides confidence in its long-term trajectory toward standardization.

### 5.4. Limitations

Our framework has several limitations: (1) Scalability: Current implementation supports up to 50 UAV nodes; larger swarms may experience increased communication overhead. (2) Non-IID Data: Our evaluation assumes an IID data distribution; real-world deployments may exhibit non-IID characteristics. (3) Hardware Constraints: CRYSTALS-Dilithium signature sizes (2.4 KB) may challenge ultra-low-bandwidth links. (4) Evolving Threats: Static model approach limits adaptability to novel zero-day attacks. (5) Privacy–Utility Tradeoff: Stronger privacy (ε < 0.5) causes significant accuracy degradation (>5%).

## 6. Conclusions

This paper presents a comprehensive quantum-resilient federated learning framework for multi-layer cyber anomaly detection in unmanned aerial vehicle (UAV) systems. By jointly integrating CRYSTALS-Dilithium post-quantum digital signatures, Byzantine-robust federated optimization, and differential privacy, the proposed framework provides robust defense against both current-day adversaries and emerging quantum-enabled attack scenarios. The core detection architecture is built on a hybrid Variational Autoencoder (VAE)–classifier model that synergistically combines unsupervised anomaly detection with supervised attack classification, thereby surpassing conventional single-paradigm detection approaches. This complementary learning strategy enables reliable identification of both known attack patterns and previously unseen (zero-day) threats within complex UAV communication environments.

Future work will address scalability to larger swarms through hierarchical federated learning, implement continual learning mechanisms to address evolving threats, and explore hardware acceleration for post-quantum cryptographic operations.

## Figures and Tables

**Table 1:** Dataset distribution by attack category.

Attack Category	Samples	Percentage	Description
Normal	77,156	55.8%	Legitimate UAV operations (Benign Flight)
GPS Spoofing	24,187	17.5%	False GPS signal injection via HackRF
GPS Jamming	17,628	12.8%	Radio frequency jamming of GPS signals
Ping DoS	10,912	7.9%	MAVLink ping flooding attack
Simulated Attacks	8377	6.0%	Gazebo-simulated attack scenarios
Total	138,260	100.0%	**-**

**Table 2:** Validation results for α selection. The bold row indicates the selected value of α used in all subsequent experiments.

α Value	Validation Accuracy (%)	F1-Score	Observation
0.2	95.34 ± 0.4	0.9512	Favors classification, weak anomaly detection
0.3	97.18 ± 0.3	0.9698	Improved balance
**0.4**	**98.67 ± 0.2**	**0.9856**	**Optimal balance (selected)**
0.5	97.94 ± 0.3	0.9778	Slight over-emphasis on VAE
0.6	96.52 ± 0.4	0.9634	Classification accuracy degraded

**Table 3:** Mathematical notation table.

Symbol	Dimension	Description
x	ℝ^48^	Input UAV feature vector
z	ℝᵈ	Latent variable in VAE
Μ_φ(x)_	ℝᵈ	Mean of latent distribution
σ^2^_φ(x)_	ℝᵈ	Variance of latent distribution
W_1_	ℝ^256×48^	First-layer weight matrix
W_2_	ℝ^128×256^	Second-layer weight matrix
W_3_	ℝ^64×128^	Third-layer weight matrix
W_4_	ℝ^5×64^	Output layer
$\hat{y}$	ℝ^5^	Predicted class probability vector
ℒ_VAE	scalar	Variational autoencoder loss
ℒ_CE	scalar	Cross-entropy loss
ℒ_hybrid_	scalar	Combined VAE + classifier loss
w⁽ᵗ⁾	model params	Global federated model
w_k_⁽ᵗ⁾	model params	Client-k local model
g_k_⁽ᵗ⁾	model params	Local gradient
α	[0, 1]	Hybrid loss weight

**Table 4:** Centralized performance of the proposed hybrid VAE–NN framework (no FL, no DP).

Metric	Value
Anomaly Detection Accuracy	98.67%
Multi-class Classification Accuracy	98.67%
Macro F1-Score	0.9856
Signature Verification Time	1.8 ms
Computational Overhead	6.8%

**Table 5:** Ablation study variants.

Variant	Components	Purpose
Baseline	NN Classifier only	Establish baseline performance
VAE-only	VAE anomaly detection	Evaluate unsupervised learning
Classifier-only	Multi-class NN	Evaluate supervised learning
VAE+Classifier	Hybrid architecture	Assess complementary benefits
+DP	Add differential privacy	Measure privacy cost
+Krum	Add Byzantine robustness	Complete framework

**Table 6:** Class-wise performance under federated learning with DP and Krum aggregation.

Attack Type	Precision	Recall	F1-Score	Support
Normal	0.9834	0.9921	0.9877	11,573
GPS Spoofing	0.9689	0.9578	0.9633	3628
GPS Jamming	0.9612	0.9489	0.9550	2644
Ping DoS	0.9567	0.9456	0.9511	1637
Simulated Attacks	0.9478	0.9334	0.9405	1257
Overall	-	-	0.9595	20,739

**Table 7:** Ablation study results.

Model Variant	Accuracy (%)	F1-Score	Improvement	Training Time
NN Classifier (Baseline)	92.23 ± 0.3	0.9198	-	2.3 h
VAE Only	94.45 ± 0.4	0.9423	+2.22%	3.1 h
Classifier Only	92.56 ± 0.3	0.9234	+0.33%	2.3 h
VAE + Classifier	98.67 ± 0.2	0.9856	+6.44%	4.2 h
+Differential Privacy	96.08 ± 0.3	0.9587	+3.85%	4.5 h
+FL + DP + Krum (Federated)	96.38 ± 0.2	0.9618	+4.15%	5.1 h

**Table 8:** Performance under Byzantine attacks.

Aggregation	Malicious Nodes	Accuracy (%)	F1-Score	Convergence
FedAvg	0%	98.67	0.9856	100 epochs
FedAvg	10%	93.56	0.9334	120 epochs
FedAvg	20%	88.23	0.8801	150 epochs
FedAvg	30%	72.45	0.7198	No convergence
Krum	0%	98.38	0.9618	105 epochs
Krum	10%	95.78	0.9556	110 epochs
Krum	20%	94.38	0.9416	120 epochs
Krum	30%	92.01	0.9178	135 epochs

**Table 9:** Cryptographic operations performance comparison.

Operation	ECDSA	CRYSTALS-Dilithium	Overhead	Quantum-Safe
Key Generation	2.3 ms	2.8 ms	+21.7%	✓
Signing	1.2 ms	1.4 ms	+16.7%	✓
Verification	1.7 ms	1.8 ms	+5.9%	✓
Signature Size	64 bytes	2420 bytes	+3681%	✓
Public Key Size	33 bytes	1312 bytes	+3875%	✓
Memory Usage	12 KB	18 KB	+50%	✓
Overall Latency *	45.2 ms	48.3 ms	+6.8%	✓

* Overall latency measured for complete federated learning round with 10 UAV node. ✓ indicates that the cryptographic scheme is quantum-safe.

**Table 10:** Privacy–Utility Tradeoff Analysis.

ε Value	Accuracy (%)	F1-Score	Privacy Level	Noise σ
0.1	82.45 ± 1.2	0.8212	Very Strong	38.7
0.5	91.34 ± 0.8	0.9098	Strong	7.74
1.0	96.26 ± 0.3	0.9608	Moderate	3.87
2.0	96.38 ± 0.2	0.9618	Weak	1.94
5.0	96.45 ± 0.2	0.9623	Very Weak	0.77
∞ (No DP)	96.67 ± 0.2	0.9645	None	0

**Table 11:** Real-time performance on UAV hardware.

Metric	Value	Requirement
Inference Latency	12.3 ms	<50 ms
Throughput	81 samples/s	>50 samples/s
CPU Usage	34%	<50%
GPU Usage	67%	<80%
Memory Usage	1.2 GB	<2 GB
Model Size	18.4 MB	<50 MB
Update Size	4.8 MB	<10 MB
Battery Impact	+8% drain	<15% increase

**Table 12:** Cyber–physical attack detection performance.

Attack Type	Intensity	Detection Rate	False Positive Rate
GPS Spoofing	10% deviation	96.2%	2.1%
GPS Spoofing	50% deviation	99.1%	0.8%
Sensor Data Injection	Subtle	94.8%	3.2%
Sensor Data Injection	Aggressive	98.7%	1.1%
Command Hijacking	MAVLink injection	97.3%	1.8%

**Table 13:** Training data sensitivity analysis.

Experiment	Configuration	Accuracy (%)	Std. Dev.
5-Fold Cross-Validation	Full dataset	98.67	±0.24
Training Size: 100%	96,782 samples	98.67	±0.20
Training Size: 70%	67,747 samples	96.89	±0.35
Training Size: 50%	48,391 samples	93.56	±0.52
Balanced Classes	Undersampled	97.34	±0.31
Temporal Split	80/20 chronological	97.28	±0.28

**Table 14:** Comprehensive comparison with related works.

Study	Year	Dataset	Accuracy	Privacy	Byzantine	Quantum	Method
Zhang [19]	2021	UAV-IDS	98.58%	✗	✗	✗	CNN
Wei [27]	2020	MNIST	97.1%	✓	✗	✗	FL-DP
Zhao [30]	2022	Custom	94.2%	✗	✗	✗	FL-DNN
Chen [32]	2017	Synthetic	95.3%	✗	✓	✗	Byz-SGD
**This Work**	**2025**	**UAV-GCS**	**98.67%**	**✓**	**✓**	**✓**	**Hybrid FL**

## Data Availability

Data available in a publicly accessible online repository: https://ieee-dataport.org/open-access/uav-attack-dataset (https://doi.org/10.21227/00dg-0d12), accessed on 9 November 2025.
